# The Intersection Between Malaria Treatment and Chemoprophylaxis and Their Potential Adverse Dermatologic Manifestations: A Narrative Review

**DOI:** 10.7759/cureus.36066

**Published:** 2023-03-13

**Authors:** Christopher S Farkouh, Michelle R Anthony, Faiza Amatul, Parsa Abdi, Qaisar Ali Khan

**Affiliations:** 1 Dermatology, Rush Medical College, Chicago, USA; 2 Pathology, University of Arizona College of Medicine, Tucson, USA; 3 Dermatology, Mercer University School of Medicine, Macon, USA; 4 Medicine, Memorial University of Newfoundland, St. John's, CAN; 5 Internal Medicine, Medical Teaching Institution (MTI) Khyber Teaching Hospital (KTH), Peshawar, PAK; 6 Internal Medicine, District Headquarters (DHQ) and Teaching Hospital, Kohat, PAK

**Keywords:** plasmodium malariae, narrative review, cutaneous manifestations, pharmacological adverse effects, malaria treatment

## Abstract

Malaria is a life-threatening parasitic disease caused by various forms of the protozoa *Plasmodium* and is transmitted by the female *Anopheles* mosquito. The parasitic infection is endemic in 90 countries, with approximately 500 million cases reported annually and an estimated annual mortality of 1.5-2.7 million individuals. Historically, the use of antimalarial drugs has been promising for the chemoprophylaxis and treatment of malaria, mitigating the annual mortality rate. Notably, these antimalarial drugs have been associated with various adverse effects, including gastrointestinal upset and headaches. However, the adverse cutaneous manifestations these antimalarial drugs may lead to are poorly documented and understood. We aim to describe the lesser-studied adverse cutaneous pathologies of malaria treatment to better educate physicians on the proper treatment of their patients.

Our narrative review describes the skin manifestations associated with specific antimalarial treatments and their associated prognoses and treatments. The cutaneous pathologies discussed include aquagenic pruritus (AP), palmoplantar exfoliation, Steven-Johnson syndrome, toxic epidermal necrolysis, cutaneous vasculitis, psoriasis, ecchymosis, and tropical lichenoid dermatitis. Further studies and vigilant documentation of the cutaneous adverse events of antimalarial drugs need to be performed and emphasized to prevent potential life-threatening adverse outcomes.

## Introduction and background

Malaria is a life-threatening disease caused by several species of the parasite *Plasmodium* and spread by the female *Anopheles* mosquito. Every year, around two billion individuals are prone to malaria infection with around 500 million cases of malaria confirmed in those at risk. With rising global temperatures in the 90 malaria-endemic countries across the globe, it is no surprise that 1.5-2.7 million people annually succumb to the virus [1]. Five known species of *Plasmodium* cause infection in humans, with *P. falciparum* infection leading to the most severe complications and mortality. *Plasmodium falciparum* is most prevalent in Africa, specifically in western and sub-Saharan Africa, where the mortality rate post infection is around 90% [1].

The most common symptom of malaria is a long-standing fever. Other common symptoms include headache, malaise, gastrointestinal pain, and muscle aches. Severe malaria cases can even lead to seizures, cerebral malaria, anemia, sepsis, renal failure, acute respiratory distress syndrome, disseminated intravascular coagulation, and pulmonary edema. Around 80% of malaria-associated deaths are due to cerebral malaria with *P. falciparum* [1]. To combat the fatal virus, antimalarial drugs have been used in recent years for both the treatment and chemoprophylaxis against malaria. Although these antimalarial drugs are generally proven effective in resolving malaria infections, adverse effects can arise with their administration, including, but not limited to, gastrointestinal upset, dizziness, persistent cough, and headaches [2]. Recent literature documenting cases of dermatologic pathologies arising in those undergoing antimalarial treatment suggests rare adverse effects of these pharmacological treatments. In focusing on treating the common systemic side effects, physicians may overlook the adverse cutaneous manifestations of antimalarial treatment as they tend to be rare and are thus understandably less analyzed.

Objectives

Our narrative review summarizes adverse cutaneous effects due to antimalarial drugs used for the treatment and chemoprophylaxis against malaria in an attempt to better prepare physicians in identifying and treating these skin manifestations.

Methods

A literature search was performed using PubMed, Scopus, and Google Scholar. Our search was focused on the various adverse skin pathologies the patients noted to have experienced after malaria treatment and chemoprophylaxis, using primarily case reports supplemented with narrative reviews and systematic reviews. Articles were selected and narratively reviewed by two reviewers. In the initial article selection process, a third reviewer was included in times of incongruity between the two primary reviewers.

## Review

Results

Aquagenic Pruritus (AP) Induced by Antimalarial Drugs

In 1981, aquagenic pruritus (AP) was established as a separate diagnosis from aquagenic urticaria [3]. AP is described as a skin disease with severe itching without lesions that typically occurs after contact with water [4]. In addition to displaying a hereditary tendency, AP is associated with primary polycythemia, polycythemia vera, juvenile xanthogranuloma, myelodysplastic syndrome, non-Hodgkin lymphoma with T-cells, hepatitis C infections, and medications such as bupropion. Nigerian young adults, for example, commonly suffer from AP caused by bathing in bodies of non-sterilized water, with a prevalence of around 23.8% [3]. Additionally, a subset of AP has been prevalent among the elderly, who are continually exposed to warm, dry, and ambient temperatures. This subset differs from classic AP because it responds exceptionally well to emollients [3].

It has been found that antimalarial drugs have the potential to induce non-aquagenic pruritus in almost 75% of patients [4]. This generalized pruritus can persist for up to 72 hours and is noted to have no proven relief from nonsteroidal anti-inflammatory drugs (NSAIDs). Antimalarial drug-induced AP was first described in 1988 after a study detailing six lupus patients undergoing dual treatment with hydroxychloroquine and chloroquine. These individuals presented with AP after 1-3 weeks of using the aforementioned drugs, suggestive of a potential correlation [4]. The users of clomipramine, tricyclic antidepressants, and bupropion have also reported incidences of drug-induced AP [3,4]. Symptoms exacerbated by water contact included a high-intensity itch (that lasted around 10 minutes, which then later decreased in intensity after several hours) and an erythematous afflicted area due to severe pruritus, ultimately leaving excoriations in the area.

A case study involving a 36-year-old non-lupus-afflicted African female detailed a lifelong history of generalized pruritus in relation to a malaria-endemic environment [4]. She was placed on frequent quinine and chloroquine doses without confirmatory malaria testing because of her pruritus. After each course of medication, the patient displayed generalized pruritus with no systemic symptoms within 30 minutes after contact with water nor after a drastic change in air temperature. Although the patient had refrained from the continual treatment of antimalarial medications, she continued to suffer from persistent, intense pruritus with water contact thereafter [3].

This diagnosis of AP can be quite challenging as AP includes no additional symptoms nor visible rash, a negative symptomatic dermographism, and a negative TempTest for cold/heat contact urticaria [3]. Additionally, blood investigations, including full blood count, thyroid function, hepatitis B and C serology, and other genetic screening blood tests, are also unremarkable, apart from an increased erythrocyte sedimentation rate (ESR). The case study notes a positive response to the administration of loratadine (10 mg twice daily) and ranitidine (150 mg twice daily). Patients were counseled on their AP and scheduled for annual blood count monitoring as they have an increased susceptibility to polycythemia vera and other myeloproliferative disorders [3].

Palmoplantar Exfoliation Due to Chloroquine

Chloroquine, although discontinued from the treatment of *P. falciparum* infection in the majority of virus-endemic regions due to the global spread of resistant parasites, still can be utilized effectively in certain instances of malaria treatment [5]. Regarding its treatment efficacy, chloroquine is known to cause mild cutaneous side effects such as pruritus, drowsiness, vision changes, bradycardia, and sudden dizziness. However, there have been instances where the medication has caused more significant adverse cutaneous events such as photosensitivity and palmoplantar exfoliation.

Nair and Patel documented an incidence of photosensitivity and palmoplantar exfoliation in a 40-year-old female five days after taking a chloroquine tablet for malaria treatment [5]. The patient experienced depigmentation of her palms and soles with exfoliation, particularly along the margin of decoloration. Her lesion was also accompanied by pruritus, believed to be exacerbated by photosensitivity. While the lesion was visibly striking, it was benign with normal complete blood count (CBC), liver function tests (LFTs), renal laboratory tests, and absent lymphadenopathy or other systemic signs. This presentation accounts for one of the most severe documented, though it was relatively non-life-threatening [5].

Unusual Cutaneous Manifestations With Mefloquine

Mefloquine is an antimalarial drug often used for chemoprophylaxis against *Plasmodium*. It is effective as an antimalarial drug as it targets multiple proteins in the parasite [6]. The most commonly reported cutaneous reactions include pruritus and maculopapular rash. Severe complications such as Steven-Johnson syndrome, toxic epidermal necrolysis, and cutaneous vasculitis have been noted to occur as a result of the drug's usage. However, various case reports have also reported unusual mefloquine-associated cutaneous manifestations such as psoriasis and ecchymosis [7-9].

The most commonly reported adverse skin reaction of mefloquine includes pruritus, with severe cases described as "an excruciating burning" [8]. The second most common manifestation is a confluent maculopapular rash, commonly noted in adults with a male predominance. Pruritus and maculopapular rash manifest around 11 days after the use of mefloquine [8] and often resolve after the discontinuation of the drug, though a minority of patients may require supportive corticosteroids and antihistamines for complete resolution.

Smith et al. described a review wherein a 66-year-old female undergoing malaria prophylaxis using mefloquine developed a blister on the lower part, which gradually transformed into ulcerating lesions involving her oral mucosa, upper and lower extremities, and back [8]. After 35 days of oral steroids and antihistamines, the patient's symptoms resolved without complication. Additionally, another case is described where a six-year-old female developed a rash that progressed to toxic epidermal necrolysis 35 days after taking prophylactic mefloquine. Unfortunately, the patient died 19 days after onset [8].

Chew and Ponampalam described a 46-year-old individual who developed ecchymosis of the trunk, waist, and buttocks with associated rectal bleeding three days after taking a prophylactic dose of mefloquine [9]. After ruling out hematological abnormalities, mefloquine was proposed as the possible etiological agent. The patient's ecchymosis resolved appropriately after stopping the usage of mefloquine [7]. Pace described a case involving a 46-year-old male with a history of well-controlled psoriasis who noticed a psoriasis flare involving his hands and feet after his third dose of mefloquine [9]. The patient's psoriasis flare improved after oral methotrexate and unspecified topical psoriasis therapy.

Potasman and Seligmann reported that the usage of mefloquine resulted in a psoriasis flare in a 50-year-old male with a history of well-controlled psoriasis limited to extensor regions [10]. After completing a one-month course of mefloquine, the patient developed anxiety, which was believed to have exacerbated his psoriatic rash, leading to its expansion to the lower extremities and trunk. Many different antimalarials have been proposed to cause psoriasis flares, but mefloquine has rarely been reported in such a context. The proposed mechanism of antimalarial drug-induced psoriasis included the Koebner phenomenon and the influence of mediators that result in epidermal proliferation [10].

Quinacrine Hydrochloride-Induced Tropical Lichenoid Dermatitis

The Allied forces used quinacrine hydrochloride for malaria prevention during World War II when quinine was unavailable after Japan invaded Southeast Asia [11]. However, quinacrine hydrochloride caused adverse skin manifestations in many of the drug recipients, with the most common drug eruption being quinacrine dermatitis [11]. Quinacrine dermatitis involves the development of a polymorphous eruption with the most common lesions being eczematoid dermatitis (80% of patients), lichenoid lesions (12% of patients), and exfoliative dermatitis (8% of patients) [11,12]. Associated pigmentary changes, atrophy of the skin, and occasional alopecia and anhidrosis were observed as well. Quinacrine hydrochloride-induced tropical lichenoid dermatitis presented as purple-to-blue hypertrophic lesions, often noted six weeks after daily oral quinacrine hydrochloride use [12]. Quinacrine hydrochloride is highly toxic to the skin because it is concentrated in keratin and is excreted through sweat, which can create the development of remittent (or chronic) lichenoid nodules and pruritic eruptions coined "post-atabrine dermatitis" [12].

Bauer further classified the permanent sequelae as inactive (first presentation) and active (recent or chronic lesions) [11]. The active lesions presented as lichen planus nodules with histological features of lichen planus and were noted to cause palmar fungating growths with histological features predominating from hypertrophic lichen planus with interspersed pseudoepitheliomatous hyperplasia. A few active lesions presented as palmar red plaques and displayed histologically as lichen planus with eczematous changes. Physicians treated the malignancies with excision or radiation therapy without complication.

## Conclusions

With the extensive benign to potentially life-threatening antimalarial-induced adverse cutaneous effects, physicians need to properly understand these events to provide competent care for their patients. Given the rare nature of these various antimalarial chemoprophylaxis- and treatment-induced adverse skin pathologies, it can be a challenge to truly encapsulate an accurate depiction of the aforementioned cutaneous presentations. Additionally, as drug metabolism varies from individual to individual, some patients may present with a more severe adverse effect than others. Lastly, had there existed extensive literature regarding antimalarial cutaneous adverse effects, further insight into the pathologies of the adverse skin effects could have been deduced from the cases presented. The development of rare but severe manifestations such as Steven-Johnson syndrome, toxic epidermal necrolysis, and cutaneous vasculitis, along with the exacerbation of chronic conditions such as psoriasis, requires further studies and extensive documentation to be performed and emphasized.
